# The Middlesex Hospital

**Published:** 1916-03-04

**Authors:** 


### THE MIDDLESEX HOSPITAL.

### SOME POINTS OF INTEREST.

At the annual meeting of the Court of Governors of this hospital, held at the hospital, Berners Street, on the 24th ultimo, an interesting discussion arose on the large excess of £4,414 in the year's expenditure over income. To meet this expenditure part of the hospital's invested funds had to be sold, the loss on the sale amounting in round figures to £273. It was significant that a woman, Mrs. Turnbull, was the only governor present who criticised the wisdom of meeting the deficiency by a sale of the hospital stock at a time when all investments were so seriously depreciated. It is further notable that the deficiency in question would not have arisen had the hospital from the outset made arrangements with the War Office, as did other hospitals where the business management is good, whereby an allowance would have been made towards the cost of treatment of wounded soldiers, the total cost of which during the year had been £6,406, of which sum no less than £5,434 had in consequence to be met out of the general funds of the hospital. We are glad to hear that more businesslike arrangements have been made with the War Office in regard to the admission of wounded soldiers during 1916. The Government will allow 4s. per day for each soldier patient received into the hospital, and 3s. per day for those treated at the Clacton branch.

### The Bland Sutton Institute.

The Bland Sutton Institute of Pathology has been completed, and laboratory investigations for the hospital have been carried out during 1915. The general accounts for 1915, as audited and submitted to the annual meeting, contained a memorandum to the effect that the grant from King Edward's Hospital Fund for London was made subject to the condition that the expenses of the Bland Sutton Institute of Pathology would be allocated in the accounts for 1915 in accordance with the scheme of apportionment to be approved by the Fund. A scheme is now being considered by the King's Fund, so that the accounts as presented were subject to any adjustment which that Fund may advise being made before publication.

### Hospital Income.

The Chairman, Sir Henry Morris, F.R.C.S., congratulated the Board on pointing out in regard to hospital income that during the war the power of the public to give is weaker than the wish to help, but the need for help remains unchanged. While recognising this, the Board continue confident that the ordinary work of the hospital, as well as that special service which it has undertaken—the care of the wounded—will not be allowed to suffer. In endorsing this opinion, we earnestly hope that the revenue shown in the accounts of Middlesex Hospital for 1916 will be found more than to justify it.

### A Hint to the Wise.

The financial results for the present year might be made more satisfactory by avoiding in future the extravagance in the form and paper adopted when printing the audited accounts, balance-sheet, returns of patients, and other documents presented to the Court of Governors, the whole of which appear to have been specially set in large type for this purpose. Some of the great hospitals are usefully adopting the plan followed by important business undertakings, which includes the preparation of a carefully drawn statement or speech giving an interesting account of the work of the institution, to be read or made by the chairman at the quarterly court and also at the annual meeting. We hope this hint will be taken in good part by the authorities of Middlesex Hospital.

